# Functional regeneration of the transected recurrent laryngeal nerve using a collagen scaffold loaded with laminin and laminin-binding BDNF and GDNF

**DOI:** 10.1038/srep32292

**Published:** 2016-08-25

**Authors:** Baoxin Wang, Junjie Yuan, Xinwei Chen, Jiafeng Xu, Yu Li, Pin Dong

**Affiliations:** 1 Department of Otolaryngology, Head and Neck Surgery, Shanghai General Hospital, Shanghai Jiao Tong University School of Medicine, Shanghai 201620, P.R. China; 2 Department of Orthopedics, Shanghai Fengxian District Central Hospital, Shanghai Jiao Tong University Affiliated Sixth People’s Hospital South Campus, Shanghai 201499, P.R. China; 3 School of Economics and Finance, Shanghai International Studies University, Shanghai 200083, P.R. China

## Abstract

Recurrent laryngeal nerve (RLN) injury remains a challenge due to the lack of effective treatments. In this study, we established a new drug delivery system consisting of a tube of Heal-All Oral Cavity Repair Membrane loaded with laminin and neurotrophic factors and tested its ability to promote functional recovery following RLN injury. We created recombinant fusion proteins consisting of brain-derived neurotrophic factor (BDNF) and glial cell line-derived neurotrophic factor (GDNF) fused to laminin-binding domains (LBDs) in order to prevent neurotrophin diffusion. LBD-BDNF, LBD-GDNF, and laminin were injected into a collagen tube that was fitted to the ends of the transected RLN in rats. Functional recovery was assessed 4, 8, and 12 weeks after injury. Although vocal fold movement was not restored until 12 weeks after injury, animals treated with the collagen tube loaded with laminin, LBD-BDNF and LBD-GDNF showed improved recovery in vocalisation, arytenoid cartilage angles, compound muscle action potentials and regenerated fibre area compared to animals treated by autologous nerve grafting (*p* < 0.05). These results demonstrate the drug delivery system induced nerve regeneration following RLN transection that was superior to that induced by autologus nerve grafting. It may have potential applications in nerve regeneration of RLN transection injury.

Causes of recurrent laryngeal nerve (RLN) injury include idiopathic disease, neck surgery, neck trauma, and malignancy^1^,^2^. RLN injury results in vocal cord paralysis, which often leads to hoarseness, dysphagia and dyspnoea and may even be life-threatening^3^,^4^. RLN injury not only reduces patients’ quality of life, but also imposes psychosocial and economic burdens. There are currently no effective methods to restore vocal cord function in patients with vocal cord paralysis^5^. End-to-end anastomosis of the transected nerve is commonly used to repair a short resection gap, and autologous nerve grafting (ANG) is used to repair a large gap. However, these methods do not lead to functional recovery^1^,^3^,^6^,^7^,^8^. Furthermore, ANG has several significant shortcomings, including the additional trauma induced by harvest of the donor nerve, limited donor sites and lengths of available grafts, impaired target organ function in the donor area, and 3-dimensional structural incompatibility between the recipient and donor nerves^9^,^10^,^11^.

Scaffolding biomaterials can be used to bridge nerve gaps and aid regeneration^12^. Desirable characteristics of these materials include low antigenicity, high cell compatibility and bioactivity, and the potential to provide an enclosed space for guiding axon migration across the nerve gap^13^,^14^,^15^,^16^,^17^. Nondegradable materials such as silicone are associated with adverse reactions due to mechanical effects or infection^18^. Although biodegradable materials can avoid these problems, the degradation products may decrease the pH, which is harmful to the surrounding tissue^19^. Collagen is an extracellular matrix component that plays an important role in neurite outgrowth^20^. Because of its abundance, biodegradability, and low immunogenicity, collagen has been widely used in peripheral nerve (PN) regeneration procedures for several years^21^,^22^,^23^,^24^,^25^. Heal-All Oral Cavity Repair Membrane is a xeno-acellular dermal matrix that is filled with collagen and dissolves in approximately 6 months. In a previous study, a xeno-acellular dermal matrix was found to be a suitable biological material because the cytotoxic effects could be eliminated by repeated irrigation^26^. Owing to its biocompatibility, Heal-All film is available in China for clinical applications with governmental approval.

Because of the complicated pathophysiological processes underlying PN injury, a single treatment method is not sufficient to achieve functional recovery, and therefore concurrent therapies should be applied^13^,^27^. Accordingly, we have established a sustained-release drug delivery system composed of a Heal-All collagen tube containing laminin and neurotrophic factors. Laminin is an important component of the PNS extracellular matrix that may affect cell myelination and spreading^12^,^28^. Glial cell line-derived neurotrophic factor (GDNF) has been reported to promote motor neuron survival and nerve regeneration after nerve injury^29^. Brain-derived neurotrophic factor (BDNF) has been shown to promote nerve regeneration and angiogenesis^30^. Research has shown that BDNF that has been fused to a laminin-binding domain (LBD-BDNF) promotes nerve recovery more effectively than BDNF alone, owing to prevention of rapid BDNF diffusion by the LBD^12^,^31^,^32^. However, the regenerative effects of a recombinant LBD-GDNF fusion protein compared to GDNF alone have not been established.

Efforts have been made to promote PN repair using biomaterials; however, such studies have achieved limited functional recovery^11^,^33^,^34^. In this study, we test a novel combinatorial repair strategy for promoting functional nerve regeneration following complete transection of the RLN in rats. We form Heal-All Oral Cavity Repair Membrane into a tube, which is fitted to the injured nerve to provide a favourable environment for nerve regeneration. We also hypothesised that this collagen tube may provide an optimal scaffold for the delivery of therapeutic substances such as neurotrophic factors. Thus, we injected the collagen tubes with laminin, LBD-BDNF and/or LBD-GDNF. This procedure represents the first use of Heal-All Oral Cavity Repair Membrane, as well as the combination of LBD-BDNF and LBD-GDNF, for the treatment of RLN injury. We propose that this drug delivery system may be useful for the prevention of misdirected reinnervation during RLN regeneration.

## Results

### Surface and SEM views of Heal-All Oral Cavity Repair Membrane

**Figure 1:**
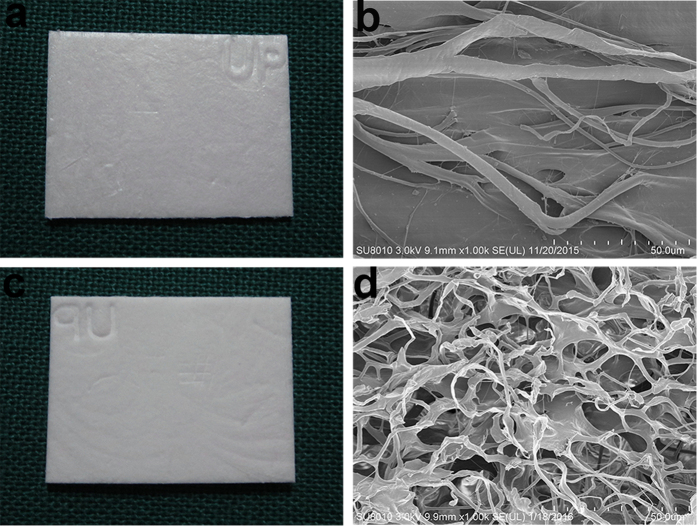
Structure of Heal-All Oral Cavity Repair Membrane. The basement membrane (**a**) and porous surface (**c**) were taken in kind. SEM images displayed the microstructure of the basement membrane surface (**b**) and porous surface (**d**).

Figure 1a,c show the surface features of dry Heal-All Oral Cavity Repair Membrane. Figure 1b shows the basement membrane surface. Figure 1d shows the organisation of the porous surface, with an average (±SD) pore size of 60 ± 40 μm.

### Bioactivity of LBD-BDNF and LBD-GDNF on laminin *in vitro*

**Figure 2:**
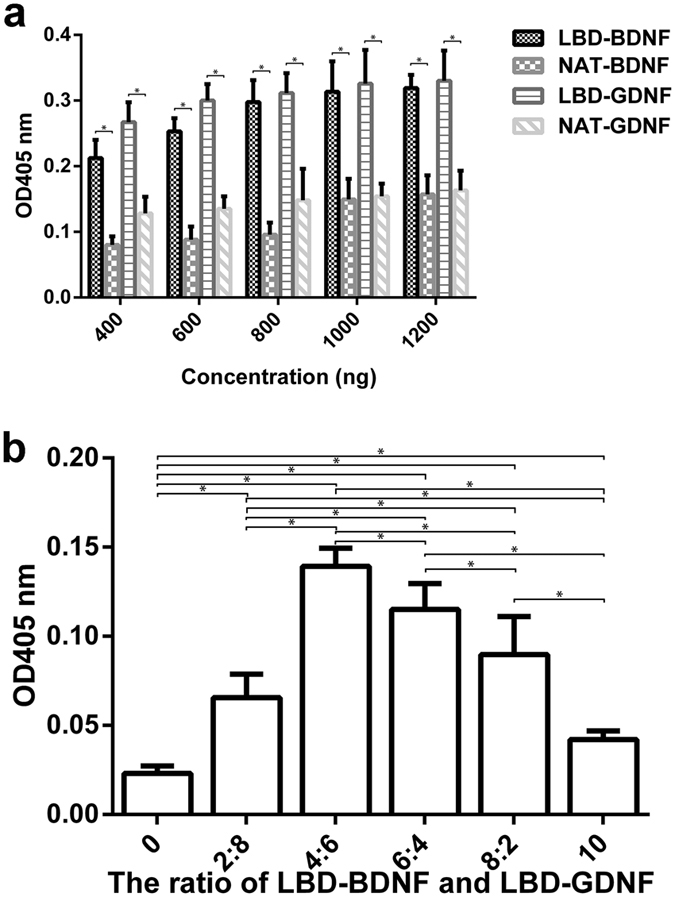
Effect of recombinant proteins on cell survival in PC 12 cells by CCK-8 assay. (**a**) More living cells were found in the neurotrophic factors with NtA, when the same quality recombinant proteins were provided. (**b**) More living cells were found in only LBD-GDNF added than only LBD-BDNF (*P* < 0.05). Whatever ratios of LBD-BDNF and LBD-GDNF mixed are preferable than single usage of LBD-BDNF or LBD-GDNF (*P* < 0.05), and the most living cells were found when the ratio of LBD-BDNF to LBD-GDNF was 2:3 (*P* < 0.05). The ratio of 0 or 10 indicates cells were cultured in the mentioned conditions without LBD-BDNF or LBD-GDNF. n = 6. *P < 0.05.

PC12 cell survival was enhanced by addition of LBD-BDNF or LBD-GDNF to laminin-coated culture wells (n = 6, *p* < 0.05; Fig. 2a). LBD-BDNF and LBD-GDNF demonstrated higher bioactivities on laminin than BDNF and GDNF. PC12 cell survival was maximised when the ratio of LBD-BDNF to LBD-GDNF was 2:3 (Fig. 2b).

### Surgical outcomes

All rats survived until the chosen endpoints with no signs of infection. No significant inflammation, dislocation, bulges or neuroma were observed surrounding the nerve ends, and the collagen tubes remained intact and did not collapse.

### Vocalisation recovery following RLN injury

**Figure 3:**
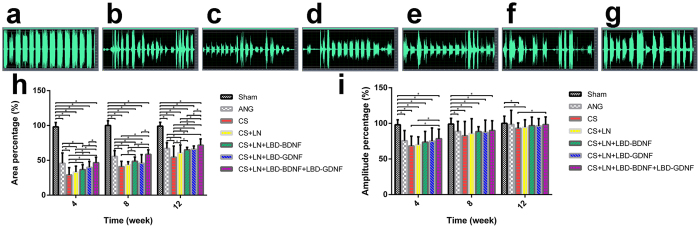
Vocalisation spectras, spectral area and amplitude percentages of different groups were shown. Vocalisation spectras were shown of the Sham (**a**), ANG (**b**), CS (**c**), CS+LN (**d**), CS+LN+LBD-BDNF (**e**), CS+LN+LBD-GDNF (**f**) and CS+LN+LBD-BDNF+LBD-GDNF (**g**) groups at 12 weeks after surgery. The Sham group exhibits a high amplitude with wide and continuous waveforms. (**h**) Spectral area percentages of different groups. The spectral area, calculated as a percentage of that in the Sham group increased over time. The spectral area percentages measured at 12 weeks after surgery were 100.0 ± 7.0%, 67.0 ± 8.2%, 54.4 ± 16.0%, 60.7 ± 11.3%, 65.1 ± 3.5%, 65.9 ± 4.6%, and 71.7 ± 9.0% of all the above group orders, respectively. The spectral area percentage of the CS+LN+LBD-BDNF group was much higher than that of the CS group at week 4, 8 and 12 week (*P* < 0.05), but much lower than that of CS+LN+LBD-BDNF+LBD-GDNF and Sham groups (*P* < 0.05). At week 12, there was no significant difference between the CS+LN+LBD-BDNF group and CS+LN+LBD-GDNF or ANG group (*P* > 0.05). The spectral area percentage of the CS+LN+LBD-BDNF+LBD-GDNF group showed no significant difference compared to that of the ANG group at 4 and 8 weeks (*P* > 0.05), while the ANG group showed significant lower compared to the CS+LN+LBD-BDNF+LBD-GDNF group at 12 weeks after surgery (*P* < 0.05). (**i**) The amplitude percentages of different groups were shown. The spectral amplitude, calculated as a percentage of that in the Sham group increased with time. The amplitude percentages of the ANG, CS+LN+LBD-BDNF, CS+LN+LBD-GDNF and CS+LN+LBD-BDNF+LBD-GDNF groups were not significantly different from that of the Sham group at 12 weeks after surgery (*P* > 0.05). From week 4 to week 12, the amplitude percentage of the CS+LN+LBD-BDNF+LBD-GDNF group was not significantly different from that of the CS+LN+LBD-BDNF or CS+LN+LBD-GDNF group (*P* > 0.05). n = 30. *P < 0.05.

Vocalisations were hoarse and disjointed in rats with RLN injury, in contrast to the continuous and shrill vocalisations of animals in the Sham group. Representative vocalisation spectra from each experimental group are shown in Fig. 3. Spectral analysis showed continuous, wide waveforms in the Sham group (Fig. 3a). Low, narrow waveforms were observed following RLN injury; however, waveform amplitude and continuity increased over time (Fig. 3i). The spectral area, calculated as a percentage of that in the Sham group, also increased with time (Fig. 3h). The mean spectral areas of the CS+LN+LBD-BDNF and CS+LN+LBD-GDNF groups were not significantly different from that of the ANG group at 12 weeks after surgery (*p* = 0.2479 and *p* = 0.5242, respectively). However, we observed a significant increase in spectral area in the CS+LN+LBD-BDNF+LBD-GDNF group compared to the ANG group (*p* < 0.05). Furthermore, only the CS and CS+LN groups showed significant differences in spectral area compared to the ANG group. At week 12, only the CS and CS+LN groups showed significant differences in spectral amplitude compared to the Sham group.

### Vocal fold movement following RLN injury

**Figure 4:**
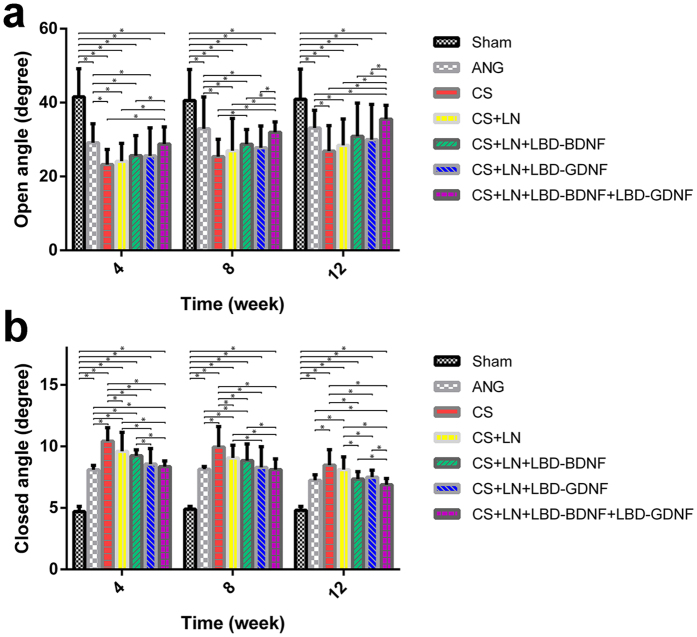
(**a**) Open and (**b**) closed angles between arytenoid cartilages during movement of the vocal fold. The arytenoid cartilages’ angles of the arytenoid cartilages improved over time. (**a**) The open angles measured 4, 8 and 12 weeks after surgery were 28.79 ± 4.63°, 31.97 ± 2.78°, 35.52 ± 3.78° of the CS+LN+LBD-BDNF+LBD-GDNF group, respectively. The open angle of the CS+LN+LBD-BDNF+LBD-GDNF group showed no significant difference compared to that of the ANG group at 4 and 8 weeks after surgery (*P* > 0.05); while the CS+LN+LBD-BDNF+LBD-GDNF group showed significant improvements compared to the ANG group at 12 weeks (*P* < 0.05). (**b**) The closed angles at week 4, 8 and 12 were 8.35 ± 0.46°, 8.11 ± 0.87°, 6.88 ± 0.51° of the CS+LN+LBD-BDNF+LBD-GDNF group, respectively. Twelve weeks after surgery, the CS+LN+LBD-BDNF+LBD-GDNF group showed markedly smaller closed angle compared to the ANG group (*P* < 0.05), although the angle remained worse than that of the Sham group (*P* < 0.05). n = 30. *P < 0.05.

Rats with RLN injury showed no motor function in the right vocal fold immediately after surgery, with a vocal fold movement score of 0. At all time points studied, no significant movement of the right vocal cord was observed in any group except for the Sham group. The maximal abduction and adduction angles of the arytenoid cartilages improved over time in all groups except the Sham group (Supplementary Fig. S1 and Fig. 4). Twelve weeks after surgery, the CS+LN+LBD-BDNF+LBD-GDNF group showed markedly improved abduction and adduction angles compared to the ANG group (*p* < 0.05), although these angles remained worse than those of the Sham group (*p* < 0.05).

### Electromyographical responses

**Figure 5:**
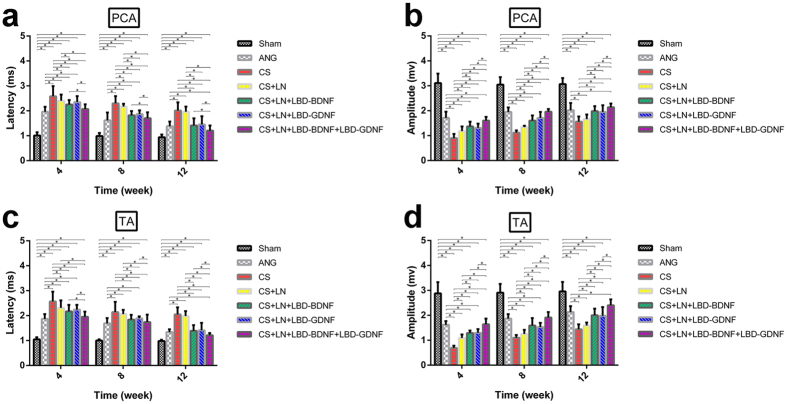
The latency and amplitude of the right PCA and TA muscles in the above groups. In all experimental groups, response latencies decreased and amplitudes increased from 4 to 12 weeks after surgery, although none of the groups showed responses at week 12 that were comparable to those of the Sham group (*p* < 0.05). (**a**) The latencies of the right PCA were significantly restored in the CS+LN+LBD-BDNF+LBD-GDNF group compared to those of the CS+LN+LBD-BDNF or CS+LN+LBD-GDNF group from week 4 to week 12 (*P* < 0.05). The latencies were shorter in the ANG group than in the CS+LN+LBD-BDNF or CS+LN+LBD-GDNF group from week 4 to week 8 (*P* < 0.05) and the latencies of the CS+LN+LBD-BDNF+LBD-GDNF group were shorter than those of the ANG group at week 12 (*P* < 0.05). (**b**) The amplitudes of the right PCA were significantly restored in the ANG group compared to those of the CS+LN+LBD-BDNF or CS+LN+LBD-GDNF group from week 4 to week 8 (*P* < 0.05). The amplitudes were much higher in the CS+LN+LBD-BDNF+LBD-GDNF group than in the CS+LN+LBD-BDNF or CS+LN+LBD-GDNF group at all time points after surgery (*P* < 0.05). (**c**) The latencies of the right TA were significantly restored in the CS+LN+LBD-BDNF+LBD-GDNF group compared to those of the CS+LN+LBD-GDNF group from week 4 to week 12 (*P* < 0.05). The latencies of the CS+LN+LBD-BDNF group showed significant differences compared to those of the CS+LN+LBD-BDNF+LBD-GDNF group, except for week 8. The latencies were shorter in the ANG group than in the CS+LN+LBD-BDNF or CS+LN+LBD-GDNF group from week 4 to week 8 (*P* < 0.05). Further, the differences were not significant between the ANG group and the CS+LN+LBD-BDNF or CS+LN+LBD-GDNF group. (**d**) The amplitudes of the right TA were significantly restored in the ANG group compared to those of the CS+LN+LBD-BDNF or CS+LN+LBD-GDNF group from week 4 to week 8 (*P* < 0.05). The amplitudes were much higher in the CS+LN+LBD-BDNF+LBD-GDNF group than in the CS+LN+LBD-GDNF or CS+LN+LBD-GDNF group at all time points (*P* < 0.05). n = 30. *P < 0.05.

EMG response latencies and amplitudes measured in the TA and PCA muscles at 4, 8 and 12 weeks after injury showed varying levels of recovery among the experimental groups (Supplementary Fig. S2 and Fig. 5). In all experimental groups, response latencies decreased and amplitudes increased from 4 to 12 weeks after surgery, although none of the groups showed responses at week 12 that were comparable to those of the Sham group (*p* < 0.05). EMG response latencies and amplitudes were significantly restored in the ANG, CS+LN, CS+LN+LBD-BDNF, CS+LN+LBD-GDNF and CS+LN+LBD-BDNF+LBD-GDNF groups compared to those of the CS group. Responses recorded from the ANG group showed no significant differences compared to those from the CS+LN+LBD-BDNF and CS+LN+LBD-GDNF groups; however, the CS+LN+LBD-BDNF+LBD-GDNF group showed significant improvements in response latency and amplitude compared to the ANG group.

### BDNF and GDNF expression in the larynx

**Figure 6:**
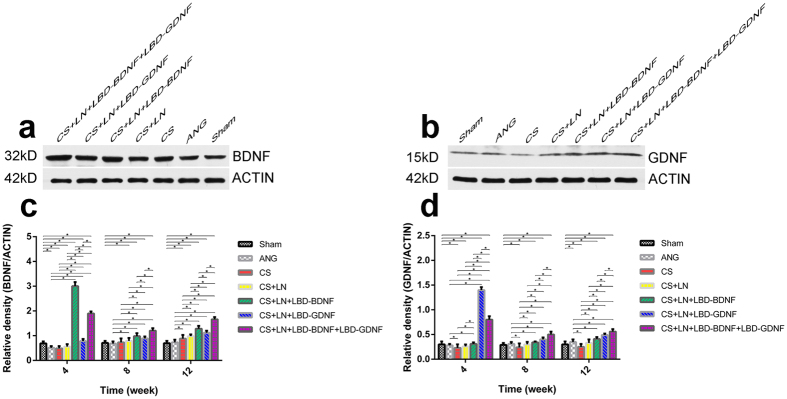
Detection of BDNF and GDNF expression in the larynx. (**a,c**) Detection of BDNF expression in the laryngeal by Western blotting analysis. (**b,d**) Detection of GDNF expression in the laryngeal by Western blotting analysis. (**c**) Expression of BDNF tended to increase from 4 to 12 weeks after surgery, except for the CS+LN+LBD-BDNF and CS+LN+LBD-BDNF+LBD-GDNF groups. BDNF expression was markedly higher in the CS+LN+LBD-BDNF, CS+LN+LBD-GDNF or CS+LN+LBD-BDNF+LBD-GDNF group than in the Sham group at all time points (*P* < 0.05). While BDNF expression in the CS+LN+LBD-BDNF group was higher than in the CS+LN+LBD-BDNF+LBD-GDNF group at week 4 (*P* < 0.05), BDNF expression in the CS+LN+LBD-BDNF+LBD-GDNF group was higher than in the CS+LN+LBD-BDNF group at weeks 8 and 12 (*P* < 0.05). (**d**) Expression of GDNF tended to increase from 4 to 12 weeks after surgery, except for the CS+LN+LBD-GDNF and CS+LN+LBD-BDNF+LBD-GDNF groups. GDNF expression was markedly higher in the CS+LN+LBD-BDNF, CS+LN+LBD-GDNF or CS+LN+LBD-BDNF+LBD-GDNF group than in the Sham group at all time points (*P* < 0.05). GDNF expression in the CS+LN+LBD-GDNF group was higher than in the CS+LN+LBD-BDNF+LBD-GDNF group at week 4 (*P* < 0.05), while this pattern was reversed at weeks 8 and 12 (*P* < 0.05). n = 30. *P < 0.05.

BDNF and GDNF expression levels in the larynx were tested by western blot analysis (Fig. 6a,b). Overall, expression of both proteins tended to increase from 8 to 12 weeks after surgery. BDNF expression was markedly higher in the CS+LN+LBD-BDNF and CS++LN+LBD-BDNF+LBD-GDNF groups than in the Sham group at 4, 8 and 12 weeks after surgery. Although BDNF expression (Fig. 6c) in the CS+LN+LBD-BDNF+LBD-GDNF group was lower than in the CS+LN+LBD-BDNF group at 4 weeks after surgery, this pattern was reversed at weeks 8 and 12. Furthermore, while GDNF expression (Fig. 6d) in the CS+LN+LBD-GDNF group was higher than in the CS+LN+LBD-BDNF+ LBD-GDNF group at 4 weeks after surgery, GDNF expression in the CS+LN+LBD-BDNF+LBD-GDNF group was higher than in the CS+LN+LBD-GDNF group at week 8 and 12.

### BDNF and GDNF expression in the RLN

**Figure 7:**
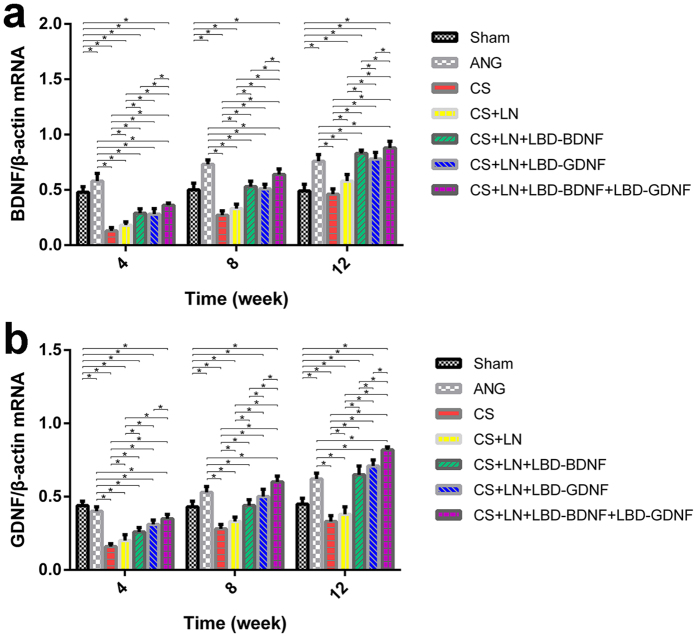
BDNF (**a**) and GDNF (**b**) mRNA expression in the right recurrent laryngeal nerve by real-time PCR. (**a**) BDNF mRNA expression increased over time in all experimental groups. Although mRNA expression in the CS+LN+LBD-BDNF group was markedly lower than in the ANG group at weeks 4 and 8 (*P* < 0.05), this pattern was reversed at week 12 (*P* < 0.05). Furthermore, BDNF expression was markedly lower in the CS+LN+LBD-BDNF group than in the CS+LN+LBD-BDNF+LBD-GDNF group at all time points (*P* < 0.05). (**b**) GDNF mRNA expression increased over time in all experimental groups. GDNF mRNA was higher in the CS+LN+LBD-GDNF and CS+LN+LBD-BDNF+LBD-GDNF groups than in the ANG group at week 12 (*P* < 0.05). Furthermore, GDNF expression was much lower in the CS+LN+LBD-GDNF group than in the CS+LN+LBD-BDNF+LBD-GDNF group at all time points after surgery (*P* < 0.05). n = 15. *P < 0.05.

RT-qPCR using mRNA isolated from the right RLN showed changes in BDNF and GDNF expression following nerve transection and experimental treatments (Fig. 7). BDNF expression increased over time in both the CS+LN+LBD-BDNF and CS+LN+LBD-BDNF+LBD-GDNF groups. Although BDNF expression in the CS+LN+LBD-BDNF group was lower than in the ANG group at week 8, this pattern was reversed at week 12 (*p* < 0.05). Furthermore, BDNF expression was higher in the CS+LN+LBD-BDNF+LBD-GDNF group than in the CS+LN+LBD-BDNF group at all time points after surgery (*p* < 0.05).

GDNF expression was higher in the CS+LN+LBD-GDNF and CS+LN+LBD-BDNF+LBD-GDNF groups than in the ANG group at week 12. Furthermore, GDNF expression was higher in the CS+LN+LBD-BDNF+LBD-GDNF group than in the CS+LN+LBD-GDNF group at all time points after surgery (*p* < 0.05).

### Remyelination analysis

**Figure 8:**
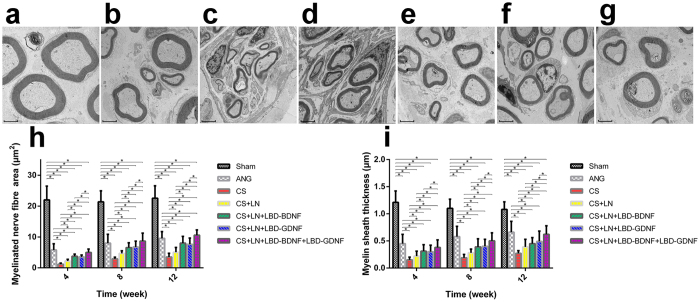
Transmission electron microscopy of the middle segment of the regenerated nerve at 12 weeks after surgery. (**a**), the Sham group; (**b**), the ANG group; (**c**), the CS group; (**d**), the CS+LN group; (**e**), the CS+LN+LBD-BDNF group; (**f**), the CS+LN+LBD-GDNF group; (**g**), the CS+LN+LBD-BDNF+LBD-GDNF group. Myelinated nerve fibre cross sectional area (**h**) and myelin sheath thickness (**i**) were quantitatively evaluated and statistically analyzed. (**h**) Myelinated nerve fibre cross sectional area of all experimental groups increased with time. The area was higher in the CS+LN+LBD-BDNF+LBD-GDNF group than in the CS+LN+LBD-BDNF or CS+LN+LBD-GDNF group at all time points (*P* < 0.05), and higher than in the ANG group at week 12 (*P* < 0.05). (**i**) Myelin sheath thickness of all experimental groups increased with time. The thickness was thicker in the CS+LN+LBD-BDNF+LBD-GDNF group than in the CS+LN+LBD-BDNF or CS+LN+LBD-GDNF group (*P* < 0.05), and there was a negligible increase in the ANG group compared to the CS+LN+LBD-BDNF+LBD-GDNF group at all time points (*P* < 0.05). Scale bar = 2 μm. *P < 0.05.

The cross-sectional area of regenerated RLN fibres and the thickness of their myelin sheaths were analysed using TEM (Fig. 8). In general, fibre cross-sectional area and myelin thickness increased over time. Both nerve fibre cross-sectional area and myelin thickness in the CS group were notably lower than in other groups. The CS+LN group also showed limited remyelination. However, significant myelin regeneration was observed in the CS+LN+LBD-BDNF, CS+LN+LBD-GDNF and CS+LN+LBD-BDNF+LBD-GDNF groups. Myelinated nerve fibre cross-sectional area was higher in the CS+LN+LBD-BDNF+LBD-GDNF group than in the ANG group at week 12. Further, we observed only a negligible increase in myelin sheath thickness in the ANG group compared to the CS+LN+LBD-BDNF+LBD-GDNF group 12 weeks after surgery. Quantification of the TEM results confirmed these observed increases in myelinated axon cross-sectional area and myelin sheath thickness in the CS+LN+LBD-BDNF+LBD-GDNF group (Fig. 8h,i).

## Discussion

Transection or crushing of PN fibres between the brain and target organs results in paralysis of the corresponding structure. Although PNs have the ability to regenerate after injury, regeneration is dependent upon the microenvironment^6^; thus, providing an appropriate microenvironment is critical for PN regeneration and functional recovery^35^. Researchers have long attempted to use neurotrophic factors, cell transplantation, tissue engineering and other treatments to promote nerve regeneration^22^,^33^,^34^,^35^,^36^; however, functional recovery remains unsatisfactory. Owing to the severe effects of RLN injury on patients’ quality of life and the economic burden on families and society, an effective method to promote axon regeneration is urgently needed.

The use of tissue-engineering technology to enhance PN repair is attracting increasing attention. In this study, we used a rat model of RLN injury to test a novel drug delivery system combining a collagen tube combined with 2 neurotrophic factor fusion proteins. Because previous research demonstrated that linear ordered structure could support a linear order guidance for spouting axons^6^, the basement membrane which exhibited approximate linear ordered structure was the interior surface of the prepared collagen tube. We used a transection model in which 5 mm of the RLN were removed; thus, our results reflect true axon regeneration, as no remaining nerve fibres were left after injury. Naturally occurring materials such as collagen, an ECM component, may provide a favourable environment for cell adhesion and migration^37^. Based on this theory, we tested the ability of a collagen scaffold to promote RLN regeneration. Previous studies show that axons and Schwann cells of the proximal PN stump enter the scaffold and then extend to the distal nerve stump^38^. However, the use of a biological scaffold alone is not sufficient to achieve complete nerve repair. Neurotrophic factors are known to modulate the differentiation, survival and regeneration of PNS neurons^39^. In order to prevent the rapid diffusion of recombinant neurotrophins in the extracellular space, researchers have incorporated neurotrophic factors into fusion proteins. For example, laminin- and collagen-binding domains have been fused to fibronectin^40^, BDNF^22^,^30^,^31^,^32^,^41^,^42^,^43^,^44^,^45^, nerve growth factor^20^,^46^,^47^,^48^ and ciliary neurotrophic factor^6^. No studies using LBD-GDNF have been published to date. Laminin and collagen IV are ubiquitous elements of basement membranes^49^. After PN injury, laminin production by Schwann cells at the injury site is upregulated to promote axonal regeneration^6^,^48^. Thus, we used laminin and LBD-fusion constructs to maintain high local concentrations of BDNF and GDNF. PC12 cell survival assays showed that LBD-BDNF and LBD-GDNF maintained higher bioactivities than BDNF and GDNF alone because of their high concentrations in laminin-coated wells. Based on these results, we conclude that the addition of LBDs increases the binding of BDNF and GDNF to laminin and allows sustained neurotrophin release.

Because the RLN controls laryngeal muscle movement, the recovery of vocal fold movement and arytenoid cartilage angles were measured as indicators of functional nerve regeneration. Recovery of motor function was not observed in all experimental groups. This pattern may be due to the severity of the PN injury used in this model, as nerve regeneration is more difficult following severe injuries^48^ and therefore motor recovery may be incomplete. Although none of the therapeutic methods tested in this study were able to restore vocal fold movement, vocalisation abilities improved over time in several groups, perhaps benefiting from the preserved tone and muscle mass of the vocal fold^50^. At 12 weeks after surgery, vocalisations in the CS+LN+LBD-BDNF+LBD-GDNF group were significantly improved compared to those in the ANG, CS+LN+LBD-BDNF and CS+LN+LBD-GDNF groups.

Axon regeneration and remyelination are critical to functional recovery following nerve injury. Previous studies have reported that facilitating the myelination of regenerated axons improves functional recovery following PN injury^51^. At 12 weeks after injury, although myelin sheath thickness was similar between the CS+LN+LBD-BDNF+LBD-GDNF and ANG groups, the CS+LN++LBD-BDNF+LBD-GDNF group showed significantly increased myelinated nerve fibre cross-sectional area compared with the ANG group, suggesting that sustained release of BDNF and GDNF may accelerate axon maturation. Consistent with this observation, CMAP recordings, which reflect the number of nerve fibres innervating target muscles^52^ and therefore provide an indicator of nerve recovery, demonstrated a similar level of functional recovery in the CS+LN+LBD-BDNF, CS+LN+LBD-GDNF and ANG groups. In contrast, while CMAP characteristics in the CS+LN+LBD-BDNF+LBD-GDNF group did not recover completely, based on comparison to the Sham group, we observed dramatic improvements in CMAP properties compared to the ANG group. Thus, although vocal fold movement did not recover, some functional connectivity between the RLN and the muscles of the larynx was restored. Overall, we conclude that our method combining the collagen tube with laminin-bound BDNF and GDNF improved axonal regeneration and maturation.

Our results demonstrate that the use of a collagen tube combined with 2 neurotrophic factors resulted in improved functional recovery compared to the use of a collagen tube with a single neurotrophin or ANG. Several mechanisms may explain this result. Firstly, the biocompatibility and biodegradability of the collagen tube make it a suitable nerve regeneration scaffold. Secondly, the high local concentrations of LBD-BDNF and LBD-GDNF due to their specific binding to laminin may promote RLN regeneration after injury by providing an improved environment for axon regrowth. Lastly, the effects of both BDNF and GDNF signalling are mediated by the Ras-mitogen-activated protein kinase and PI3 kinase pathways^53^,^54^; this overlap may explain why nerve regeneration and functional recovery were significantly improved in the CS+LN+LBD-BDNF+LBD-GDNF group compared to the CS+LN+LBD-BDNF and CS+LN+LBD-GDNF groups in our experiments.

We also show increased expression of BDNF and GDNF in the RLN and larynx of rats treated with collagen tubes combined with neurotrophic factors. BDNF has been reported to prevent the death of Schwann cells^55^, which also express and secrete neurotrophins to promote axonal regeneration^56^. This phenomenon may help to explain the increased expression of endogenous BDNF and GDNF in the RLN, as measured by RT-qPCR. However, the increased expression of neurotrophic factors in the larynx, as revealed by western blot analysis, includes both endogenous and exogenous proteins. It is likely that, in the CS+LN+LBD-BDNF and CS+LN+LBD-BDNF+LBD-GDNF groups, levels of exogenous BDNF lessened and those of endogenous BDNF increased over time. At week 4, BDNF expression in the CS+LN+LBD-BDNF group was higher than that in other groups, likely owing to the migration of exogenous BDNF to the laryngeal muscles. The decrease in BDNF expression in the CS+LN+LBD-BDNF group compared to CS+LN+LBD-BDNF+LBD-GDNF group at week 8 may be due to a significant decrease in exogenous BDNF and an increase in endogenous BDNF. The further increase in BDNF expression in the above groups at week 12 may be explained by an increase in endogenous BDNF, as well as decreased retrograde transport efficiency, leading to BDNF accumulation in the laryngeal muscles^5^. The significant increase in BDNF expression in the CS+LN+LBD-BDNF+LBD-GDNF group compared to the CS+LN+LBD-BDNF group at week 12 may be due to the synergistic effects of LBD-BDNF and LBD-GDNF by promiting the effects of BDNF.

The promotion of neurite growth and growth cone formation by laminin^6^ may explain the superior nerve regeneration observed in the CS+LN group compared to the CS group. However, our results show that a collagen scaffold and laminin alone is not sufficient to promote nerve recovery. Neurotrophic factor application is important to nerve regeneration; furthermore, the combination of LBD-BDNF and LBD-GDNF more effectively enhanced RLN regeneration compared to use of either neurotrophin alone.

Functional recovery is one of the most important therapeutic goals following RLN injury. At present, ANG remains the clinical gold standard of treatment for RLN injury^57^,^58^, an autologous nerve is the most optimal repair material because of its comparatively low cost and risk of side effects. These advantages have previously outweighed the disadvantages of ANG when used in clinical practice. However, our data show that the combination of a collagen scaffold with laminin and LBD-BDNF or LBD-GDNF results in a similar level of functional recovery to that obtained with ANG; furthermore, the addition of both LBD-BDNF and LBD-GDNF achieved superior results to those of ANG. Our method eliminates the need for a second surgery to obtain a donor nerve or to remove the conduit, which may increase the risk of infection^7^. This technique also maintains therapeutic concentrations of BDNF and GDNF at the injury site, which could reduce the necessary dosages of BDNF and GDNF and avoid undesirable systemic side effects in clinical application. Taken together, our results indicate that this drug delivery system facilitates axon outgrowth following RLN injury. In addition, our system uses an off-the-shelf material that can be used to create tubes of different diameters to suit different nerves.

We have created a biodegradable collagen conduit loaded with laminin for the delivery of LBD-BDNF and LBD-GDNF to sites of PN injury, where they exert synergistic effects on nerve regeneration. In the present study, this system induced nerve regeneration following RLN transection that was superior to that induced by autologous nerve grafting. These results demonstrate that the drug delivery system is strikingly effective in nerve regeneration of RLN transection injury. In the future, this procedure may be a promising therapeutic method for the treatment of peripheral nerve injuries.

The present study has several limitations. Firstly, our study was conducted over 12 weeks using small groups of rats; thus, long-term studies with larger numbers of animals will be required to verify our results. We speculate that, over time, nerve regeneration in rats treated with the CS+LN+LBD-BDNF+LBD-GDNF system may approach normal function. Secondly, it is impossible for a single animal model to reproduce all characteristics of human RLN injury. Thus, future studies should attempt to replicate our results using other animal models.

## Methods

### Preparation of recombinant proteins

LBD-BDNF and LBD-GDNF were constructed as described previously for LBD-BDNF^32^. Briefly, human BDNF and GDNF DNA was inserted into the pET-LBD and pET-28a expression vectors (Novage, San Diego, CA), which were then transformed into Escherichia coli BL21 (DE3). Fusion protein expression was induced with 1 mM isopropyl β-D-thiogalactopyranoside. Proteins were purified from inclusion bodies using nickel chelate affinity chromatography (Amersham Bioscience, Uppsala, Sweden). LBD-BDNF and LBD-GDNF were diluted with phosphate-buffered saline (PBS) to a concentration 266.67 mg/L.

### Preparation of collagen tubes

Heal-All Oral Cavity Repair Membrane (SFDA Certified No. (2015): 3460386) was purchased from Yantai Zhenghai Biotechnology Co., LTD. The film was coated with gold, and surface features were analysed on a Hitachi SU8010 scanning electron microscope (Hitachi, Tokyo, Japan). The film was hydrated in sterile saline until fully transparent and softened with no bubbles. We then used a metal core to roll the film into tubes 7 mm long with a 0.6 mm inner diameter. Tubes were sutured with 10-0 fibre sutures.

### *In vitro* bioactivity assays

BDNF, LBD-BDNF, GDNF, and LBD-GDNF were added to separate wells of a laminin-coated 96-well plate and incubated at 4 °C for 12 h. The plates were then washed to remove any unbound neurotrophic factors, and PC12 cells were seeded at a density of 3 × 10^3^ cells/well. After 48 h in culture, cell survival was measured using a Cell Counting Kit-8 assay (Dojindo, Kumamoto, Japan). As a negative control, cells were cultured without recombinant proteins under the same conditions.

The bioactivity of different proportions of LBD-BDNF and LBD-GDNF on laminin was also tested. LBD-BDNF and LBD-GDNF at ratios of 1:4, 2:3, 3:2, and 4:1 (total protein, 1,000 ng) were added to a laminin-coated 96-well plate. PC12 cell culture and survival measurements were performed as described above. As controls, cells were cultured under the same conditions with only one of the recombinant proteins.

### Animals

Two hundred and ten (210) male Sprague-Dawley rats (235 ± 15 g) were purchased from the Shanghai Laboratory Animal Center of the Chinese Academy of Science for use in the present study. All of the protocols were in accordance with the guidelines of the Institutional Animal Care and Use Committee of Shanghai Jiao Tong University and were approved by the Animal Experimental Committee of Shanghai General Hospital affiliated with the Shanghai Jiao Tong University School of Medicine. Care was taken to minimise pain throughout the study.

### Surgical procedure

Following intraperitoneally administered anaesthesia with ketamine hydrochloride (75 mg/kg) and xylazine hydrochloride (10 mg/kg), telescopic videolaryngoscopy (BF-1T160; Olympus Co., Tokyo, Japan) was performed to examine vocal fold motion during breathing. For RLN transection, a vertical midline cervical incision was made, and the larynx and trachea were visualised. The right RLN was then carefully separated from the tracheo-oesophageal groove (Supplementary Fig. S3), and 5 mm of the right RLN was removed.

The animals were divided into 7 groups, each receiving a different treatment. In the ANG group, the 5 mm-long RLN segment was re-attached in the reverse orientation using 10-0 fibre sutures^9^. In the collagen scaffold (CS) group, the 2 ends of the nerve were inserted 1 mm into the collagen tube, which was filled with a mixture of 22.5 μl of matrigel (used to increase the adhesive quality of the solution) and 7.5 μl of PBS. In the CS+laminin (CS+LN) group, a mixture of 22.5 μl of matrigel, 3.75 μl of laminin and 3.75 μl of PBS was injected into the conduit. In the CS+LN+LBD-BDNF and CS+LN+LBD-GDNF groups, a mixture of 22.5 μl of matrigel, 3.75 μl of laminin and 3.75 μl of LBD-BDNF or LBD-GDNF was injected into the conduit. In the CS+LN+LBD-BDNF+LBD-GDNF group, a mixture of 22.5 μl of matrigel, 3.75 μl of laminin, 1.5 μl of LBD-BDNF and 2.25 μl of LBD-GDNF was injected into the conduit, with a concentration ratio of the 2 fusion proteins equalling 2:3. In the sham (Sham) group, the RLN was exposed without transection or further treatment. In all groups, the muscle layers and skin were sutured separately. Telescopic videolaryngoscopy was repeated to confirm the success of the operation, as determined by the elimination of right vocal fold movement. Assessments were performed at 4, 8 or 12 weeks after surgery (n = 10 rats per group and time period).

### Measurement of vocalisation

At 4, 8, and 12 weeks after surgery, 3 vocalisation measurements at 8-hour intervals were performed for each rat. For each measurement, vocalisations were recorded over a 30-s period using a voice recorder while stimulating the right hind limb using sponge forceps in a quiet environment; Vocalisations were imported into Cool Edit Pro v. 2.1 software (Adobe Systems, Inc., USA). From each 30 s recording, one 10 s period was chosen for analysis using Photoshop CC v. 14.0 software (Adobe Systems, Inc., USA). Calculations were carried out as follows:

% Area of spectrum = Area of postoperative rats/Area of rats in Sham group

% Amplitude of spectrum = Amplitude of postoperative rats/Amplitude of rats in Sham group^59^.

### Measurement of vocal fold movement and arytenoid cartilage angles

At 4, 8, and 12 weeks following surgery, rats were anaesthetised as described above and videolaryngoscopy was performed to record vocal fold movement during spontaneous breathing. We used movement scores to evaluate vocal cord functional recovery^60^. Scoring was performed separately by two different individuals who were blinded to the treatment group of each animal in order to avoid subjective bias. A 3rd investigator’s opinion was obtained in cases where the 2 scores differed. The angles between the arytenoid cartilages were measured using images of maximal adduction and maximal abduction, and 3 images each of maximal adduction and maximal abduction were evaluated for each animal.

### Neurofunctional analysis

In order to assess neurofunctional recovery in the rats after surgery, electromyography (EMG) of the thyroarytenoid (TA) and posterior cricoarytenoid (PCA) muscles was performed. Animals were anaesthetised and the right RLN was exposed, as described above. The recording electrode was passed through the cricothyroid membrane and carefully extended upward and outward to contact the TA muscle or to penetrate the posterior aspect of the lower third of the thyroid cartilage to contact the PCA muscle. For each muscle, compound muscle action potentials (CMAPs) were recorded with the stimulating electrode applied to the proximal end of the collagen tube, using a PowerLab computer-assisted EMG machine. The nerve was stimulated until no further change was observed. Three waveforms were recorded for each rat. Response latencies and maximum amplitudes were measured.

### Western blotting

The larynx which contained all laryngeal muscles was flash-frozen in liquid nitrogen and homogenized in ice-cold RIPA lysis buffer (50 mM Tris pH 7.4, 150 mM NaCl, 1% NP-40, 0.5% sodium deoxycholate, 0.1% sodium dodecyl sulphate). After incubation on ice for 30 min, homogenates were centrifuged at 12,000 × g for 10 min at 4 °C. Equivalent amounts of proteins were separated on sodium dodecyl sulphate-polyacrylamide gels and transferred to polyvinylidene difluoride membranes (Millipore, USA). The membranes were incubated with a 1:1000 dilution of rabbit polyclonal antibodies against BDNF or GDNF (both from Santa Cruz Biotechnology) followed by HRP-conjugated secondary antibodies. Proteins were detected using enhanced chemiluminescence reagents (Pierce, Rockford, IL).

### Real-time quantitative PCR

Trizol Reagent (Invitrogen, USA) was used to extract total RNA from right RLN samples (n = 5 rats per group and time period). Reverse transcription was performed using RevertAid First Strand cDNA Synthesis Kit (Thermo, USA). The resulting cDNA was used for real-time quantitative PCR (qPCR; Applied Biosystems, USA) using specific primers for BDNF and GDNF. The real-time qPCR program was as follows: 95 °C for 10 min, followed by 40 cycles of 95 °C for 15 s and 60 °C for 60 s. Similar amplification efficiencies for the target genes and the β-actin reference gene were verified. Relative expression levels of BDNF and GDNF were calculated using the delta-delta Ct method. Primers used are listed in Supplementary Table 1.

### Transmission electron microscopy

Regenerated RLN samples (n = 5 rats per group and time period) were fixed in a 2.5% glutaraldehyde solution at 4 °C and then washed 3 times in 0.1 M PBS (pH 7.2). Samples were post-fixed in 1% OsO_4_ for 2 h, followed by 3 washes in PBS. The specimens were dehydrated in graded acetone solutions, embedded in epoxy resin and polymerised at 60 °C for 24 h. Ultra-thin (70 nm) sections were then cut from the center to the distal end using a Leica EM UC6 ultramicrotome and stained with uranylacetate and citrate. Transmission electron microscopy (TEM) images were recorded using a Tecnai G2 20 TWIN microscope (FEI, Hillsboro, OR, USA). Myelin sheath thickness and myelinated nerve fibre cross-sectional area were evaluated using Image-Pro Plus v. 6.0 software.

### Statistical analysis

All data are expressed as mean ± standard deviation (SD). The student’s t-test was used to analyse paired samples; the one-way analysis of variance (ANOVA) with Tukey’s multiple comparison test was used to analyse multiple comparisons using SPSS v. 20.0. *P* values less than 0.05 were considered significant.

## Additional Information

**How to cite this article**: Wang, B. *et al*. Functional regeneration of the transected recurrent laryngeal nerve using a collagen scaffold loaded with laminin and laminin-binding BDNF and GDNF. *Sci. Rep.* **6**, 32292; doi: 10.1038/srep32292 (2016).

## Supplementary Material

Supplementary Information
