# Supplementary material for: Functional regeneration of the transected recurrent laryngeal nerve using a collagen scaffold loaded with laminin and laminin-binding BDNF and GDNF
Source: Sci Rep. 2016 Aug 25;6:32292. doi: 10.1038/srep32292 (PMC4997630; doi:10.1038/srep32292)
Supplement: Supplementary Information [file srep32292-s1.docx]

**Functional regeneration of the transected recurrent laryngeal nerve using a collagen scaffold loaded with laminin and laminin-binding BDNF and GDNF**

Baoxin Wang^a,^, Junjie Yuan^b,^, Xinwei Chen^a,^, Jiafeng Xu^c^, Yu Li^a^, Pin Dong^a,*^


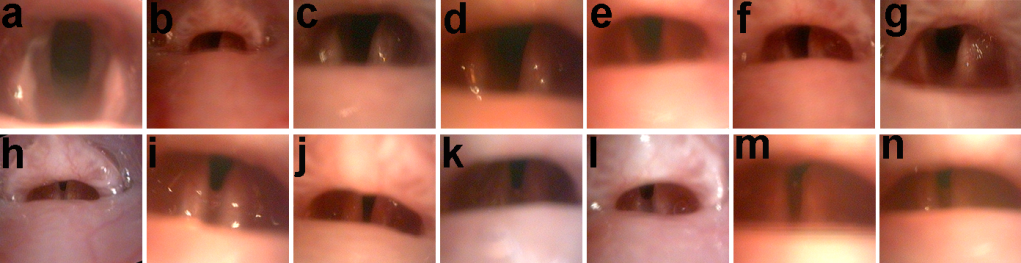


Fig. S1 Laryngoscopic image of the Sham (a), ANG (b), CS (c), CS+LN (d), CS+LN+LBD-BDNF (e), CS+LN+LBD-GDNF (f) and CS+LN+LBD-BDNF+LBD-GDNF (g) groups at maximal abduction (A) and maximal adduction (B) at 12 weeks after surgery.


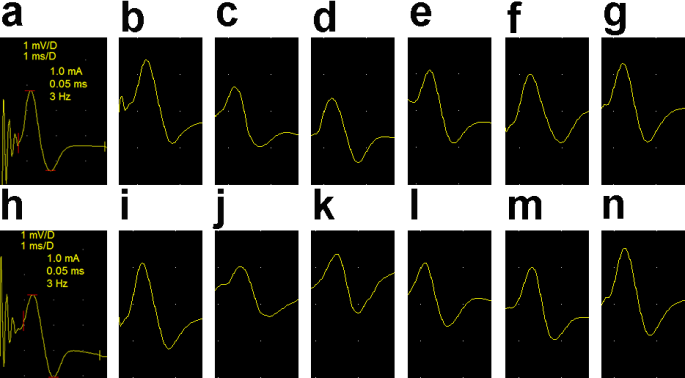


Fig. S2 EMG waveform of the Sham (a), ANG (b), CS (c), CS+LN (d), CS+LN+LBD-BDNF (e), CS+LN+LBD-GDNF (f) and CS+LN+LBD-BDNF+LBD-GDNF (g) groups at 12 weeks of the right PCA (A) and TA (B) muscles after surgery, respectively.


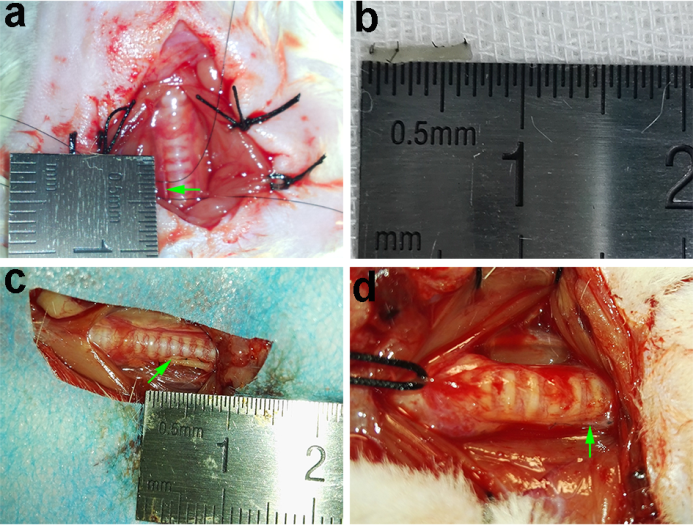


Fig. S3 The procedures of the recurrent laryngeal nerve surgery. (A) The right recurrent laryngeal nerve (green arrow) was exposed. (B) The longitudinal photo of the collagen tube, with 7 mm long and 0.6 mm inner diameter. (C) The transected nerve was bridged by the collagen tube (green arrow), and matrigel was injected into the lumen, which prevent the conduit from collapse or deformation. (D) The gross view of implanted collagen tube (green arrow) at 12 weeks postoperatively.

Table S1 PCR genes primers sequences

| Primer name | Primer sequences（5'-3') |
| --- | --- |
| R-β-actin-F | TGCTATGTTGCCCTAGACTTCG |
| R-β-actin-R | GTTGGCATAGAGGTCTTTACGG |
| R-BDNF-F | TTGATGAGACCGGGTTCCCT |
| R-BDNF-R | GTCCGTGGACGTTTGCTTCTT |
| R-GDNF-F | GGCTATGAAACCAAGGAGGAAC |
| R-GDNF-R | ATACATCCACACCTTTTAGCGG |
